# Interkingdom Gene Transfer of a Hybrid NPS/PKS from Bacteria to Filamentous Ascomycota

**DOI:** 10.1371/journal.pone.0028231

**Published:** 2011-11-29

**Authors:** Daniel P. Lawrence, Scott Kroken, Barry M. Pryor, A. Elizabeth Arnold

**Affiliations:** School of Plant Sciences , The University of Arizona, Tucson, Arizona, United States of America; University College Dublin, Ireland

## Abstract

Nonribosomal peptides (NRPs) and polyketides (PKs) are ecologically important secondary metabolites produced by bacteria and fungi using multidomain enzymes called nonribosomal peptide synthetases (NRPSs) and polyketide synthases (PKSs), respectively. Previous phylogenetic analyses of fungal NRPSs and PKSs have suggested that a few of these genes were acquired by fungi via horizontal gene transfer (HGT) from bacteria, including a hybrid *NPS/PKS* found in *Cochliobolus heterostrophus* (Dothideomycetes, Ascomycota). Here, we identify this hybrid gene in fungi representing two additional classes of Ascomycota (*Aspergillus* spp., *Microsporum canis*, *Arthroderma* spp., and *Trichophyton* spp., Eurotiomycetes; *Chaetomium* spp. and *Metarhizium* spp., Sordariomycetes) and use phylogenetic analyses of the most highly conserved domains from NRPSs (adenylation (A) domain) and PKSs (ketoacyl synthase (KS) domain) to examine the hypothesis that the hybrid *NPS7/PKS24* was acquired by fungi from bacteria via HGT relatively early in the evolution of the Pezizomycotina. Our results reveal a unique ancestry of the A domain and KS domain in the hybrid gene relative to known fungal NRPSs and PKSs, provide strong evidence for HGT of the hybrid gene from a putative bacterial donor in the Burkholderiales, and suggest the HGT event occurred early in the evolution of the filamentous Ascomycota.

## Introduction

Lateral or horizontal gene transfer (HGT) is defined as the transfer of a genetic element from one organism to another without vertical descent [1]. HGT between prokaryotes is well documented and occurs frequently through conjugation, the uptake of exogenous DNA, and virus-mediated transduction [2], [3]. HGT between eukaryotes has been documented in protists, fungi, and plants [4]–[9]. Increasingly, studies are showing that some genes residing in the genomes of extant eukaryotes were acquired via horizontal transfer from bacteria [10]–[14]. For example, molecular phylogenetic analyses have revealed that an isopenicillin-N-synthase gene was transferred horizontally from a beta-lactam-producing prokaryote to the filamentous Ascomycota, with beta-lactam production now observed in some species of *Penicillium* and *Aspergillus* [15]. Similarly, functional screens combined with phylogenetic analyses have shown that a β-glucuronidase gene was transferred from bacteria to fungi [16]. From the earliest endosymbioses and related genomic incorporation that led to the evolution of eukaryotes [17] to events that appear to be recent in evolutionary time [6], [17], prokaryote-to-eukaryote gene transfers represent a fundamental and intriguing aspect of eukaryote biology.

Evidence consistent with HGT may include high similarities between DNA or amino acid sequences in phylogenetically distant taxa, discontinuous distribution of a genetic element among members of a lineage, inconsistencies in GC content and/or codon bias across a genome, and/or presence or absence of introns [4], [18], [19]. However, the “gold standard” (sensu [1]) for detecting HGT is phylogenetic incongruence, which manifests as gene trees that conflict with the understood organismal phylogeny. Caveats for explaining such discordances exist, such as different evolutionary rates between genes or species, interspecific hybridization, ancestral gene duplication and gene loss, and mistaken comparison of paralogous genes [1], [4]. However, with careful analyses the dynamics of genes that interweave among evolutionary lineages, the frequency of HGT among different functional classes of genes, and their implications for the genetic diversity of recipient clades can be inferred. For example, phylogenetic studies have suggested that genes involved in highly complex interactions with other genes are less likely to be transferred than those with simpler interaction complexes [20], [21]. Similarly, genes required for primary metabolism are less prone to transfer than genes not required for survival under normal growth conditions (i.e., genes associated with secondary metabolism) [18]. De novo acquisition of secondary metabolite genes may open previously inaccessible niches for a recipient if they confer a selective advantage [2], [21], [22]. Such genes also may be lost after transfer if they no longer confer a selective benefit [23].

Bacteria and fungi are prolific producers of diverse secondary metabolites with varied roles: virulence factors that enable pathogens to colonize hosts, signaling molecules that respond to biotic and abiotic stimuli, pigments that protect against ultraviolet radiation, and antibiotics [24]–[26]. One class of secondary metabolites produced by numerous bacteria and fungi are nonribosomal peptides (NRPs), small-peptide secondary metabolites synthesized by multidomain enzymes called nonribosomal peptide synthetases (NRPSs). NRPSs consist of at least three domains: the adenylation domain (A) is responsible for selecting and activating specific amino acids that will be incorporated into the growing peptide by the condensation domain (C), which is responsible for peptide bond formation [27]. The thiolation or peptidyl carrier protein domain (T or PCP) acts as a swinging arm that transports substrates between the active sites of the domains for chain elongation and modification [28]. Important fungal NRPs include the antibiotic penicillin, which is produced by various species of *Penicillium* and *Aspergillus*, and the immunosuppressive drug cyclosporine produced by *Tolypocladium inflatum* [29]. Fungi also use NRPs as virulence factors or effectors, as in HC-toxin from *Cochliobolus carbonum*, a maize pathogen, and AM-toxin from *Alternaria alternata*, a pathogen of apple [30].

Similarly, polyketides (PKs) are bacterial and fungal secondary metabolites produced by large multidomain proteins called polyketide synthases (PKSs) [26], [31], [32]. Important PKs from fungi include T-toxin from *Cochliobolus heterostrophus* race T, which caused massive crop loss of maize in the 1970s [33], the carcinogen aflatoxin from *Aspergillus flavus* and closely related species [34], and pharmaceuticals such as lovastatin, a cholesterol-lowering statin from *Aspergillus terreus* [26]. Type I bacterial and fungal PKSs operate iteratively, with the catalytic site of each domain used repeatedly to produce a PK [14]. In addition to optional domains that determine the final PK structure, all type I PKSs have a minimum of three domains analogous to those found in NRPSs [35]: ketoacyl synthase (KS), acyl transferase (AT), and acyl carrier protein (ACP, also known as phosphopantetheine (PP)).

Evolution of NRPSs and PKSs in bacteria and fungi has been of keen interest in recent years [7], [14], [36], in part because of growing evidence that they have a complex evolutionary history. For example, a previous phylogenetic analysis by Kroken et al. (2003) revealed that two PKS sequences from *Co. heterostrophus* (PKS24 and PKS25) and 6-methylsalicylic acid synthase (6-MSAS) type PKS KS domain sequences from other fungi cluster with bacterial PKS KS sequences, with support from the Kishino-Hasegawa test suggesting that these genes are not of fungal origin. Moreover, the A domain from the NRPS portion and KS domain from the PKS portion of a hybrid NRPS/PKS from the fungus *Co. heterostrophus* were nested within large bacterial clades with moderate to strong support [24], [36], congruent with the hypothesis of bacterial-to-fungal HGT. Our exploratory BLAST analyses revealed that twelve additional species within the Pezizomycotina representing two additional classes contain a homolog to the *Co*. *heterostrophus* hybrid *NPS7/PKS24*. These observations suggest an HGT event may have occurred earlier in the evolution of the Pezizomycotina than was previously recognized. However, the phylogenetic relationships, frequency among related taxa, and evolutionary origins of the hybrid NPS/PKS gene in these fungi are not known.

The goal of this study was to evaluate the evolutionary history of the *Co*. *heterostrophus* hybrid *NPS7/PKS24*, and putative homologs in other fungal taxa, in a comparative phylogenetic framework. Specifically, we use phylogenetic analyses of the most highly conserved domains from NRPSs (A domain) and PKSs (KS domain) to examine the hypothesis that the hybrid *NPS7/PKS24* was acquired from bacteria via HGT relatively early in the evolution of the Pezizomycotina. Our results reveal the distinctiveness of the NRPS A domain and PKS KS domain in the hybrid NRPS/PKS protein relative to known NRPSs and PKSs in fungi, provide strong evidence for HGT of the hybrid gene from a putative bacterial donor in the Burkholderiales, and suggest a HGT event earlier in the evolution of the filamentous Ascomycota than was recognized previously.

## Results

Eighteen new sequences were generated of the hybrid *NPS/PKS* from diverse species of *Chaetomium* and *Aspergillus,* including four isolates of *Chaetomium globosum* (NRRL 1870, 6296, 25144, and 29170), three currently recognized species of *Chaetomium* (*Ch*. *rectum* NRRL 25293, *Ch*. *carpinum* NRRL 1867, and *Ch*. *olivaceum* NRRL A-14615), three unidentified species of *Chaetomium* (NRRL A-2583, A-22357, and A-23409), three isolates of *A*. *niger* (NRRL 326, 328, and 334), and five isolates representing four species of *Aspergillus* (*A*. *ficcum* NRRL 364 and 372, *A*. *foetidus* NRRL 341, *A*. *phoenicis* NRRL 4851, and *A*. *tubingensis* NRRL 4875) (Table S1). Phylogenetic analysis of GenBank sequences also confirmed homologs of the hybrid NRPS/PKS in *Microsporum canis*, four *Trichophyton* species, three *Arthroderma* species, and two *Metarhizium* species. (Tables S2, S3).

### NRPS A domain genealogy

**Figure 1:**
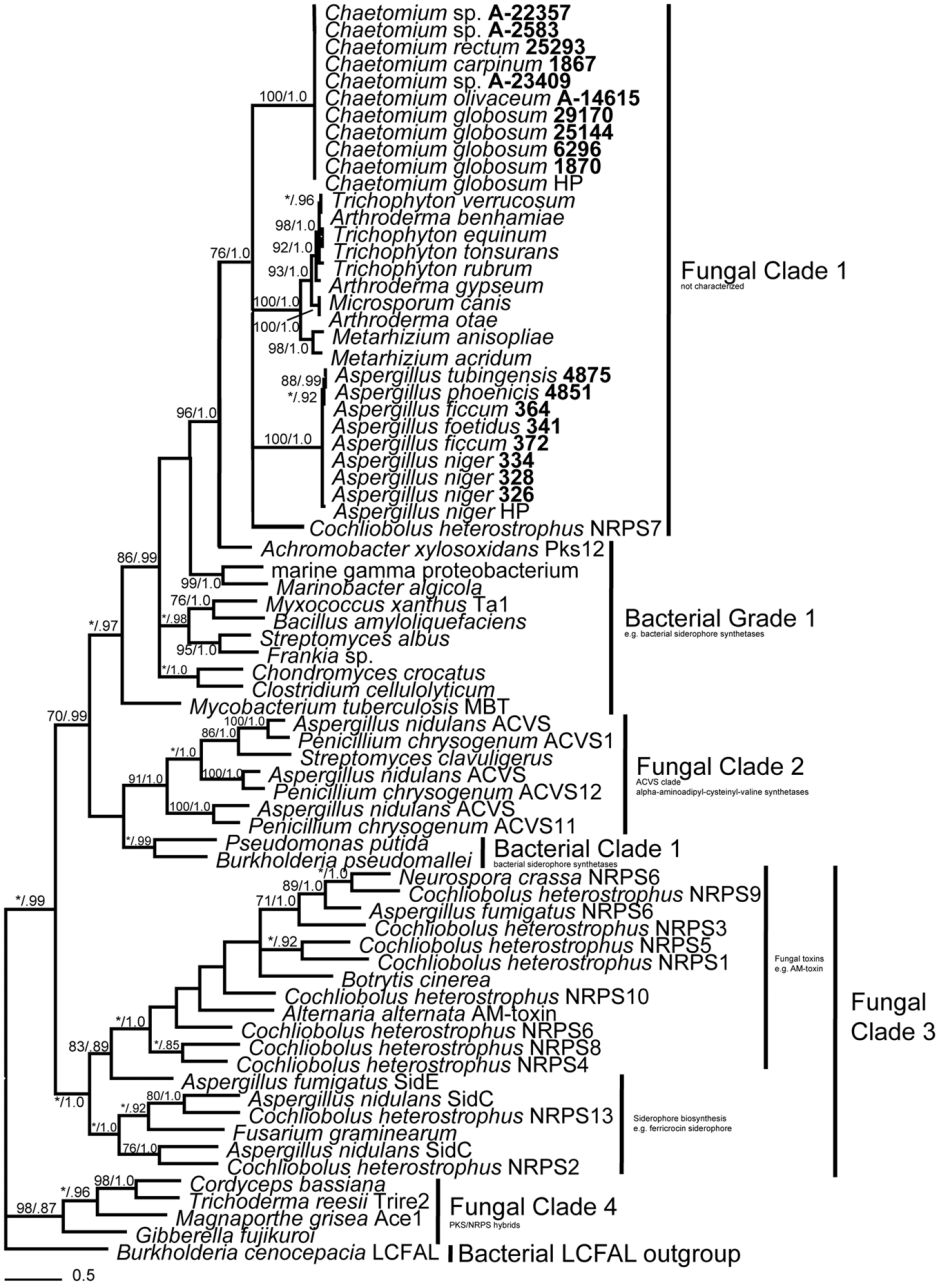
Relationship of bacterial and fungal NRPS A domains. Majority-rule consensus tree from an unconstrained Bayesian analysis based on an amino acid alignment of the NRPS A domain. Support values represent maximum parsimony bootstrap ≥70% (left) and Bayesian posterior probabilities ≥0.70 (right). Taxon numbers in bold represent sequences obtained in this study.

Phylogenetic analyses indicate a bacterial origin of the NRPS A domain in the hybrid *NPS7/PKS24* homologs from fungi examined here. Bayesian and maximum parsimony analyses of NRPS A domains place bacterial sequences (Bacterial Grade 1 and Bacterial Clade 1) with strong support among well-supported clades of fungal sequences (Fig. 1). Fungal Clade 1, obtained from amplicons of the hybrid NPS/PKS gene in this study and the sequences from *Trichophyton* spp., *Arthroderma* spp., *Metarhizium* spp., and *M. canis* from GenBank, is subtended by Bacterial Grade 1, with which it forms a strongly supported, monophyletic group sister to Fungal Clade 2. A hybrid NRPS/PKS from the bacterium *Achromobacter xylosoxidans* (Burkholderiales) is strongly supported as sister to Fungal Clade 1. Fungal Clade 1 is distantly related to non-hybrid fungal NRPS A domain sequences (Fungal Clade 3) and to A domain sequences from Fungal Clade 4 that contain PKS/NRPS hybrids (with PKS preceding NRPS as compared to Fungal Clade 1 with NRPS preceding PKS). Fungal Clade 1 shows strong structure on the basis of fungal genera and does not conflict with most known class-level relationships of fungi [37], except that *Metarhizium* spp. and *Chaetomium* spp. (Sordariomycetes) do not cluster together. The remaining fungal NRPS A domain sequences cluster into a monophyletic group that consists of fungal ACVS genes that have been shown to have arisen from bacteria via HGT [38]–[41] and is circumscribed as Fungal Clade 2.

We tested the null hypothesis that the phylogenetic history of the hybrid NRPS A domain was consistent with a fungal origin by implementing phylogenetic analyses using two types of constraint trees, here termed a “monophyly” constraint and “backbone” constraint. The monophyly constraint contains all taxa but is not fully resolved, whereas the backbone constraint enforces the placement of constrained taxa while permitting unconstrained taxa to assort across the tree according to the optimality criterion of the search [42], [43]. Tree quality scores and topologies from constraint analyses were compared against unconstrained analyses to determine the conflict imposed by forcing topologies to follow expected evolutionary histories in the absence of HGT.

**Figure 2:**
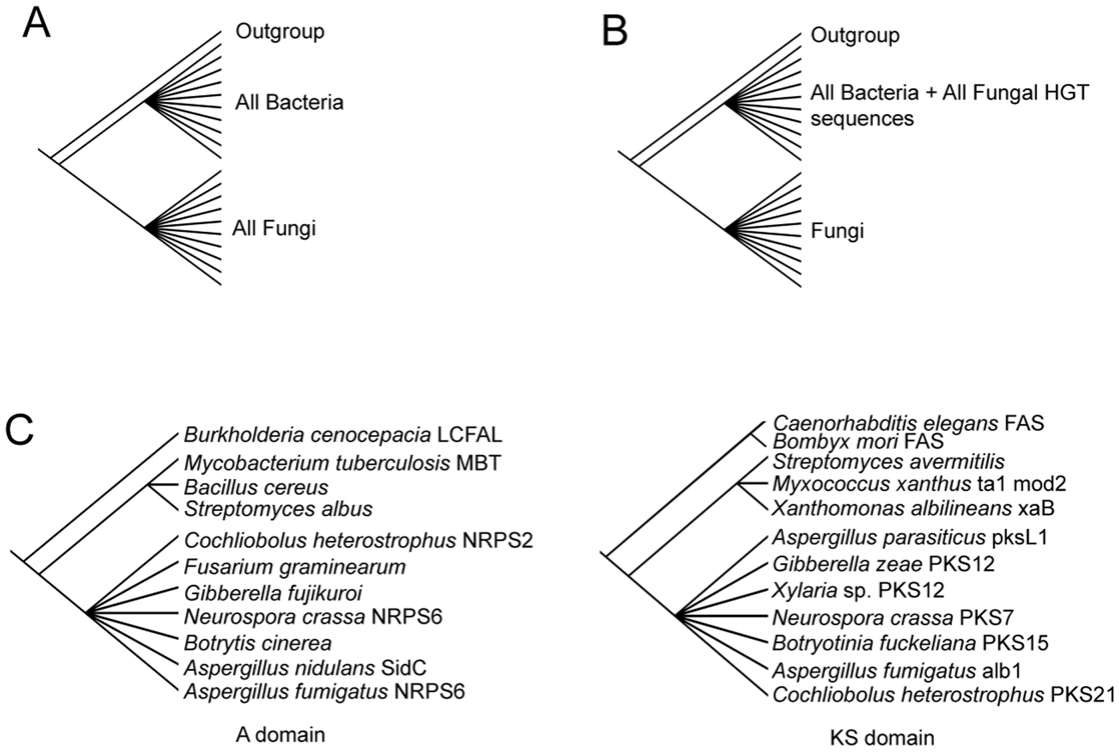
Constraints implemented for assessing topological conflict with unconstrained analyses of bacterial and fungal NRPSs and PKSs. (A) Monophyly constraint of bacterial and fungal sequences as reciprocally monophyletic polytomies sister to the outgroup; (B) monophyly constraint of bacterial sequences, fungal sequences previously identified as having undergone HGT from bacteria, and hybrid NRPS/PKS fungal sequences as a monophyletic polytomy reciprocal to a monophyletic polytomy of all other fungal sequences that together are sister to the outgroup; (C) backbone constraint of a subset of bacterial and fungal sequences as monophyletic polytomies sister to the outgroup.

Four constraint analyses were conducted. First, we constrained all bacterial sequences and all fungal sequences into reciprocally monophyletic polytomies as shown in Fig. 2A. This analysis yielded 64 equally -most parsimonious trees in which the NRPS/PKS hybrid was sister to fungal sequences (Fig. S1) rather than to bacterial sequences as in the unconstrained analysis. However, constrained trees were substantively longer (7341 steps vs. 7249 steps), had lower consistency- and retention indices, and comprised statistically different topologies relative to the unconstrained analyses of the same dataset (Table 1, A domain: constraint A).

**Table 1:** Tree statistics (tree length, consistency- and retention indices) for phylograms obtained from constrained and unconstrained maximum parsimony analyses, and results of statistical comparison of constrained vs. unconstrained topologies (asterisks indicate significant differences given alpha  = 0.05).

	Tree Length		Consistency Index		Retention Index		*P* values		
Dataset	Constrained	Unconstrained	Constrained	Unconstrained	Constrained	Unconstrained	KH^a^	TT^b^	WS^c^
A domain: constraint A	7341	7249	0.470	0.476	0.533	0.544	<0.0001*	<0.0001*	<0.0001*
A domain: constraint B	7255	7249	0.476	0.476	0.544	0.544	0.4314	0.4344	0.3317
A domain: constraint C	7255	7249	0.476	0.476	0.544	0.544	0.4314	0.4344	0.3317
KS domain: constraint A	5627	5564	0.484	0.490	0.679	0.686	<0.0001*	<0.0001*	<0.0001*
KS domain: constraint B	5564	5564	0.490	0.490	0.686	0.686	1.0000	1.0000	1.0000
KS domain: constraint C	5564	5564	0.490	0.490	0.686	0.686	1.0000	1.0000	1.0000

a Kishino-Hasegawa.

b Templeton Test.

c Winning-Sites.

Constraints are shown in Figure 2.

Second, we constrained all bacterial sequences, all fungal sequences previously identified as derived from bacteria via HGT [38]–[41] (Fig. 1, Fungal Clade 2 ACVS sequences; Table S4a), and all NRPS/PKS hybrid fungal sequences (see Fig. 1, Fungal Clade 1) as a polytomy sister to a monophyletic polytomy of the remaining fungal sequences (Fig. 2B). This analysis produced 178 equally -most parsimonious trees of 7255 steps (Fig. S2) with consistency- and retention indices similar to those of the unconstrained analysis. Their topologies were not significantly different from those of the unconstrained analysis (Table 1, A domain: constraint B).

In the third analysis, a backbone constraint was implemented by constraining only 11 sequences that included representative bacteria (3 sequences) and fungi (7 sequences) as reciprocally monophyletic polytomies sister to the outgroup (1 sequence) (Fig. 2C, A domain). This analysis produced 129 equally -most parsimonious trees of 7255 steps (Fig. S3) with identical consistency- and retention indices to those of the unconstrained analysis of the same dataset. The topologies were not significantly different from those of the unconstrained analysis (Table 1, A domain: constraint C).

In the fourth analysis, this constraint (Fig. 2C, A domain) was used in a maximum likelihood test of topology as implemented in RAxML [44] using the Shimodaira-Hasegawa test (SH-test). The topology resulting from the constrained analysis of the A domain (likelihood score -32005.936010) did not differ significantly from the unconstrained tree topology of the same dataset (likelihood score −31964.171758; *P*>0.05).

### PKS KS domain genealogy

**Figure 3:**
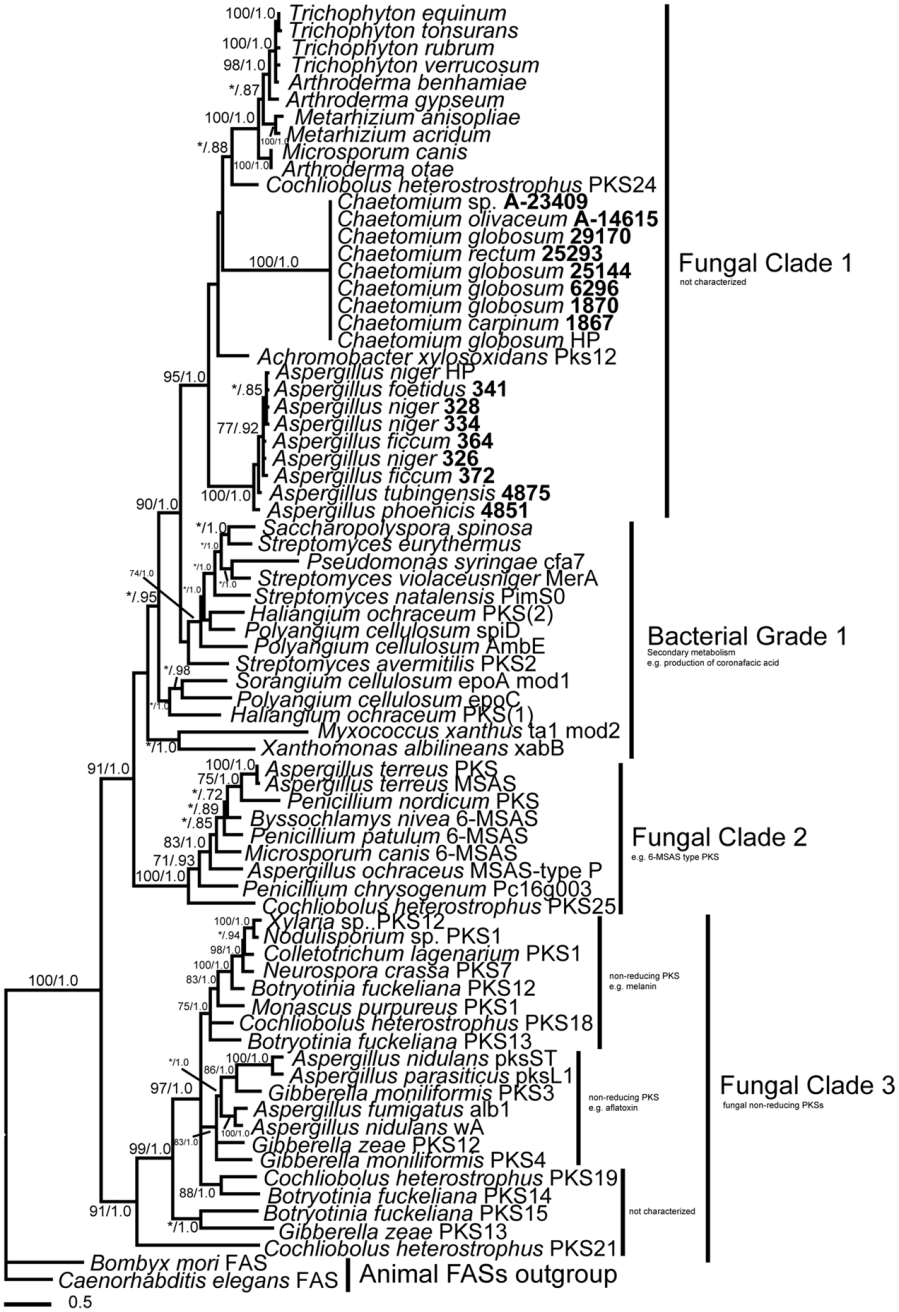
Relationship of bacterial and fungal PKS KS domains. Majority-rule consensus tree from an unconstrained Bayesian analysis based on an amino acid alignment of the PKS KS domain. Support values represent maximum parsimony bootstrap ≥70% (left) and Bayesian posterior probabilities ≥0.70 (right). Taxon numbers in bold represent sequences obtained in this study.

Phylogenetic analyses also suggest a bacterial origin of the PKS KS domain in the hybrid *NPS7/PKS24* homologs (Fig. 3). Bayesian and maximum parsimony analyses placed the hybrid KS domain (Fungal Clade 1) with strong support within a grade of bacterial sequences (Bacterial Grade 1). Fungal Clade 1, obtained from amplicons of the hybrid NPS/PKS gene in this study (Table S1) and the sequences from *Co*. *heterostrophus*, *M. canis*, *Trichophyton* spp., *Arthroderma* spp., and *Metarhizium* spp. from GenBank, is distantly related to non-hybrid fungal KS domain sequences from fungi (Fungal Clade 3). The KS domain sequence from *Achromobacter xylosoxidans* (Burkholderiales) is nested within Fungal Clade 1 with strong support. Fungal Clade 2 consists of 6-MSAS type PKSs that have been shown to have arisen in fungi via HGT from a bacterial source [14], [24]. Reconstruction of the hybrid KS domain sequences (Fungal Clade 1) shows strong structure on the basis of fungal genera. However, relationships within the clade are in partial conflict with known class-level relationships of the Pezizomycotina [37], as *Metarhizium* spp. do not cluster with the remaining Sordariomycetes.

We tested the null hypothesis that the phylogenetic history of the PKS KS domain was consistent with an origin within the fungi by using the four constrained analyses previously described. The first analysis implemented the constraint of reciprocally monophyletic polytomies of bacterial and fungal sequences (Fig. 2A) to yield 262 equally -most parsimonious trees with the hybrid NRPS/PKS sequences forming a clade sister to other fungal KS domain sequences (Fig. S4). These trees had a longer tree length (5627 steps vs. 5564 steps), lower consistency- and retention indices, and significantly different topologies relative to the unconstrained analysis of the same dataset (Table 1, KS domain: constraint A).

In the second analysis, all bacterial sequences, all fungal sequences previously identified as having undergone HGT [24] (Fungal Clade 2 6-MSAS sequences, Fig. 3; Table S4b, supplementary material) and hybrid NRPS/PKS fungal sequences (Fig. 3, Fungal Clade 1) were constrained as a polytomy sister to a monophyletic polytomy consisting of the remaining fungal sequences (Fig. 2B). This analysis yielded 249 equally -most parsimonious trees (Fig. S5, ) with identical tree lengths, consistency- and retention indices, and no statistical differences in topology as compared to the unconstrained KS domain analysis (Table 1, KS domain: constraint B).

In the third analysis 12 representative sequences were constrained, with representative bacteria (3 sequences) and fungi (7 sequences) represented as reciprocally monophyletic polytomies sister to the outgroup (2 sequences) (Fig. 2C, KS domain). This analysis produced 253 equally -most parsimonious trees of 5564 steps (Fig. S6,) with identical consistency- and retention indices to those of the unconstrained analysis. The topologies were not significantly different from those of the unconstrained analysis (Table 1, KS domain: constraint C). Implementation of the SH-test in RAxML [44] showed that the resulting topology (likelihood score - 27196.553090) did not differ significantly from the topology resulting from unconstrained analysis (likelihood score -27201.992695; *P*>0.05).

## Discussion

This study examined the phylogeny of a hybrid NRPS/PKS protein from *Co*. *heterostrophus* and homologs discovered in additional fungi by specifically exploring relationships between the NRPS A domain and the PKS KS domain and similar fungal and bacterial sequences. Previous phylogenetic studies suggested that these domains were acquired via horizontal gene transfer from an unknown bacterial source [24], [36]. However, the previous studies examined the phylogeny of each domain separately and had insufficient taxon sampling to estimate the donor or relative timing of the HGT event. The current study examines the phylogenetic history of both the A domain and KS domain as a unit and demonstrates that both domains of the hybrid NRPS/PKS protein have a similar phylogenetic history. Moreover, the results of this study are consistent with an ancient history of the intact hybrid gene in fungi, and reveal a specific bacterial lineage as the putative ancestral donor. Taken together, results of this study provide perhaps the most convincing evidence to date for HGT from bacteria to fungi of a hybrid NPS/PKS gene.

Our data revealed substantial phylogenetic distance between the hybrid A and KS domains and previously known, non-hybrid NRPS A and PKS KS domain sequences from fungi (Fig. 1 and 3). This is consistent with an independent origin relative to non-hybrid NRPSs and PKSs in the Pezizomycotina, and to the hybrid PKS/NRPSs of Fungal Clade 4 shown in Fig. 1. These new findings demonstrate that the occurrence of the hybrid homologs of *NPS7/PKS24* within the filamentous Ascomycota is much more widespread than was previously known and suggest an acquisition event early in the evolution of the Pezizomycotina before the radiation of the Leotiomyceta.

Phylogenetic analyses of the A and KS domains from *Co*. *heterostrophus* hybrid NRPS7/PKS24 and newly identified homologs in diverse Pezizomycotina reveal a closer evolutionary relationship with bacterial NRPSs and PKSs than with fungal NRPSs and PKSs (Figs. 1 and 3). This suggests acquisition via HGT from a bacterial lineage, supported statistically by the constraint analyses (Fig. 2 and Table 1).

Two additional lines of evidence further support the hypothesis of a non-fungal origin of the hybrid *NPS7/PKS24*. First, the NPS/PKS hybrid gene in *Co*. *heterostrophus* (7.6 kb), *Aspergillus niger* (5.6 kb), *Trichophyton* spp. and *Microsporum canis* (7.4 kb), do not contain introns. This contrasts with many other NPS and PKS genes in the Pezizomycotina and has been used previously to argue for a bacterial origin of 6-MSAS type PKS genes (see Schmitt and Lumbsch 2009). Interestingly, the NPS/PKS hybrid gene in *Chaetomium globosum* (7.4 kb) contains six introns of unknown provenance. The third *Chaetomium* intron sequence downloaded from the Broad Institute (http://www.broadinstitute.org/annotation/genome/chaetomium_globosum), shares 74.57% and 72.88% amino acid sequence identity to fragments of *Co*. *heterostrophus* and *A*. *niger* KS domains, respectively, revealing that the intron was misannotated. All other introns from the *Ch*. *globosum* sequence produce no significant BLAST hits in GenBank and do not translate into mature amino acid sequences without internal stops.

**Figure 4:**
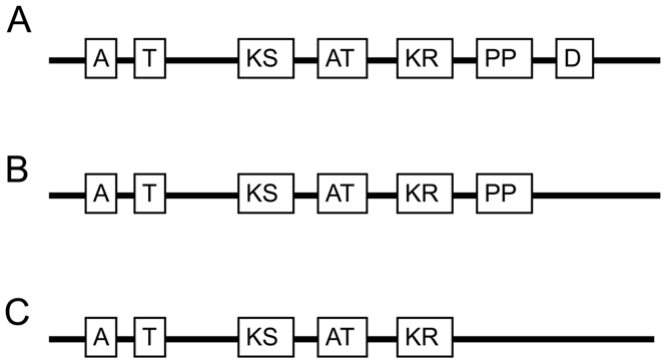
Proposed protein domain organization of NRPS7/PKS24. (A) *Co*. *heterostrophus*; (B) homolog in *A*. *niger*; (C) homolog in *Ch*. *globosum*. Adenylation (A), thiolation (T), ketoacyl synthase (KS), acyl transferase (AT), ketoreductase (KR), phosphopantetheine (PP), and dehydrogenase (D) are indicated as boxes.

Second, the common organization of hybrid NPS and PKS genes in most fungi studied to date is in the opposite orientation: PKS precedes NPS, as in *Magnaporthe grisea* ACE 1, rather than NPS preceding PKS. Figure 4 depicts the proposed domain organization of NRPS7/PKS24 from *Co*. *heterostrophus* (Ascomycota: Pezizomycotina: Dothideomycetes) and homologs identified in *Aspergillus niger* (Eurotiomycetes) and *Chaetomium globosum* (Sordariomycetes). The orientation of the hybrid NPS/PKS we examined is known mainly from bacterial genomes and putative representatives from *Aspergillus* spp. All other known hybrid genes from Dikarya and remaining fungal clades exhibit the PKS/NPS orientation [35], [36], [45].

Although independent acquisition of the hybrid NPS/PKS gene in three fungal classes (Sordariomycetes, Eurotiomycetes, and Dothideomycetes) is possible, a single acquisition of the hybrid NPS/PKS gene early in the evolution of the filamentous Ascomycota, coupled with subsequent vertical descent, lineage-specific diversification, and loss in many lineages is more parsimonious. This is concordant with the hypothesized evolution of 6-MSAS type PKS genes from lichen-forming fungi proposed by Schmitt and Lumbsch (2009), and with the common observation that secondary metabolite genes are only maintained if they confer a selective advantage [14], [23], [46]. Moreover, the relationships within Fungal Clade 1 in Figs. 1 and 3 show no significant conflict in topology based upon the Kishino-Hasegawa, Winning-Sites, and Templeton tests (*P* = 0.1497, 0.1680, and 0.3449, respectively), revealing congruent phylogeny for each domain and suggesting that the hybrid NPS/PKS gene has evolved as a single unit since its initial fungal inheritance. Recent vertical descent of the NPS/PKS hybrid gene within genera (Fungal Clade 1 in Fig. 1 and 3) is strongly suggested by high support values for the *Aspergillus* and *Chaetomium* clades of both domains. Further screening of genomic data or PCR coupled with more sensitive methods such as cloning are needed to more precisely evaluate patterns of gene loss, and to verify the lack of the hybrid NPS/PKS gene in related taxa such as *Aspergillus* Section *Flavi* and close relatives of other fungi that possess this gene.

Perhaps most importantly, this study revealed that the hybrid NRPS/PKS sequences from *Achromobacter xylosoxidans* (Burkholderiales) is sister to Fungal Clade 1 in Fig. 1 and nested within Fungal Clade 1 in Fig. 3, both with strong support. The placement and support for the close evolutionary relationship of *A*. *xylosoxidans* and Fungal Clade 1 suggests that the putative bacterial donor of the fungal NPS/PKS hybrid gene may be an ancestor of *A*. *xylosoxidans* or a closely related species within the Burkholderiales.

We have provided support for HGT from bacteria to fungi of the A and KS domains of the hybrid *NPS7/PKS24* in *Co. heterostrophus* and homologs identified in additional fungal taxa. Moreover, we revealed that both the A and KS domains have similar phylogenetic histories within fungi, suggesting the gene was acquired as a hybrid unit in early fungal lineages. By using constraint analyses we were able to show that a fungal origin of the hybrid NPS/PKS gene can be rejected by tests of topological hypotheses of constrained and unconstrained analyses. Finally, we have identified the putative donor lineage as belonging to the Burkholderiales with the sequence from *Achromobacter xylosoxidans* clustering with the fungal hybrid NRPS/PKS amino acid sequences with strong support in analyses of both domains. Future functional analyses of this hybrid NPS/PKS gene will elucidate the gene product, which is expected to be a partially reduced PK with a single amino acid decoration [24], and the roles that this secondary metabolite may play within the metabolome of these fungi.

## Materials and Methods

### Fungal cultures, DNA extraction, and PCR

Thirty-eight isolates representing 23 species of *Aspergillus* (sections *Nigri* and *Flavi*) and 71 isolates representing 28 species of *Chaetomium* were obtained from the USDA (Peoria, Illinois; Table S1). Each was transferred under aseptic technique to sterile potato dextrose broth and incubated at 25°C at 120 rpm for seven to ten days. Mycelium was filtered with sterile mira cloth, rinsed with sterile, distilled deionized water, and lyophilized for 48 hr. Total genomic DNA was extracted from lyophilized mycelium and stored at −40°C [42].

Degenerate primers for PCR were designed in MEGA 4.0 [47] by translating nucleotide sequences of *Cochliobolus heterostrophus NPS7/PKS24*, *Aspergillus niger* CAK42046 , and *Chaetomium globosum* XM_001224326 (obtained from GenBank) to amino acids prior to alignment with *Co. heterostrophus* NRPS7/PKS24, *A. niger* unnamed protein product AM270324, and *Ch. globosum* hypothetical protein CHGG_05113. We then converted the amino acid sequences back to the nucleotide sequences to identify degenerate primer pairs. We also designed *Aspergillus*- and *Chaetomium*-specific primers (Table 2) in MacVector 10.6 using nucleotide sequences mentioned above.

**Table 2:** PCR primers designed for this study, annealing temperature, and fragment length.

Primer Name	Primer Sequence (5′–3′)	Annealing Temperature	Fragment Length
PKS24F2^a^	TGGAAYHTKTAYGGCCCGAC	55°C	974 bp
PKS24R2^a^	CCBGGYAGYCKGCAHGCCAT		
PKS24F3^a^	ATGGCDTGCMGRCTRCCVGG	55°C	558 bp
PKS24R3^a^	GCCATRTCRCACTCSCCYTG		
PKS24F4^a^	TGGMRACTSTGGMAGAGCTGGGG	55°C	834 bp
PKS24R4^a^	CGCTGRAAKGCRTABGTVGGYAGCT		
ANF3^b^	TCGGTCACAATGTCGTTTCCC	59.3°C	769 bp
ANB7^b^	ATCTGTAGAATGGTCGGCGTCG		
ANF11^b^	TCTTGCCTTCTCCTGGTTTGC	59.5°C	720 bp
ANB11^b^	CGCCATTAGCATTTGAGTGAGC		
ANF1^b^	TGATAATGCGAGGTTGCCTTTG	58.9°C	968 bp
ANB28^b^	TGACGATAGGTAGCGTTTGCTCC		
ANF2^b^	TCGCAATCCTGTCCTGTTCG	59°C	779 bp
ANB21^b^	TTCGCATCACACCCCATAGC		
ANF1^b^	AACATCGCTACAGCCACCAAAC	57.9°C	471 bp
ANB12^b^	CAGAGCATCGGAAAGTGAGATAGG		
CGF2^c^	TACCAAGATGCTGGACGCCTTCAC	62.2°C	741 bp
CGB2^c^	ACAGGTGCCCATTGACGATG		
CGF1^c^	TCAAGCCGTTTGGTTGTCGC	60.1°C	611 bp
CGB2^c^	CAAGTTCAGTCCAGGATGCTCG		
CGF4^c^	ACGGGGTCACCACAACGAAAAC	61.8°C	960 bp
CGB7^c^	GCAACCAACCACCAAGTTATGC		
CGF1^c^	CATAACTTGGTGGTGGTTGC	60.3°C	439 bp
CGB6^c^	GCCTCACAAACTGCTCGTCAATC		
CGF1^c^	CACAGTTCAGGGGCATAAAGAATG	60.9°C	762 bp
CGB1^c^	TAGCACCAGGGGACACTCAACAAG		

a Degenerate primers were designed from *Co*. *heterostrophus*, *A*. *niger*, and *Ch*. *globosum* GenBank sequences.

b Primers designed from *A*. *niger* GenBank accession CAK42046 for PCR amplification of *Aspergillus* spp.

c Primers designed from *Ch*. *globosum* GenBank accession XM_001224326 for PCR amplification of *Chaetomium* spp.

PCR amplifications were performed with 2 µl of genomic DNA, 12.5 µl of 2X Master Mix (Fermentas), and 8.5 µl of nuclease free sterile water for a final reaction volume of 25 µl. PCR programs were as follows: initial denaturation at 96°C for 4 min; 40 cycles of 94°C for 30 s, 55°C for 30 s (with adjustments shown in Table 2), and 72°C for 1 min; and a final extension at 72°C for 5 min. PCR products were visualized on 1% agarose gel stained with ethidium bromide to ensure proper amplicon size. Fungal isolates in bold in Table S1 produced positive PCR amplicons.

### DNA sequencing and contig assembly

Cleanup, normalization, and bidirectional sequencing of PCR products was conducted in Applied Biosystems 3730 DNA Analyzer at the Genomic Analysis and Technology Core (GATC) at The University of Arizona. Chromatograms were edited in MacVector version 10.6 using the assembler package, and contigs were assembled using phred and phrap [48], [49]. Consensus sequences have been submitted to GenBank under accession nos. JN688002-JN688019. Assembled sequences were submitted to BLAST searches of GenBank using BLASTx to determine the correct reading frame and translated into amino acid sequences. Introns within *Chaetomium* sequences were excluded from analysis by aligning nucleotide sequences with the coding DNA sequence available from NCBI to obtain mature amino acid sequences.

### Phylogenetic analyses

The NRPS A domain and PKS KS domain of the *Co*. *heterostrophus* NRPS7/PKS24 protein [24] were individually aligned to the non-redundant database using BLASTp, with hits restricted to bacteria and fungi only. Results were used to construct a neighbor-joining tree in NCBI for each domain to identify the most closely related sequences. Ten to twenty bacterial and fungal NRPS and PKS sequences that clustered closely with *Co*. *heterostrophus* (Tables S2-, S3) were aligned to previously published alignments of amino acids [24], [36] using the profile alignment tool in CLUSTALX with default settings. Newly acquired amino acid sequences from *Chaetomium* and *Aspergillus* (above) were aligned by this method followed by manual adjustment where necessary. The sequence from *Burkholderia cenocepacia* LCFAL was used as the outgroup in the A domain analyses [36]. Two sequences from metazoans (*Caenorhabditis elegans* FAS and *Bombyx mori* FAS) were used as the outgroup in the KS domain analyses [24]. Alignments have been submitted to TreeBase under accession S11936.

The amino acid alignment for each domain was analyzed separately using maximum parsimony (MP) and Bayesian analyses. For the former, heuristic searches with 1000 random sequence additions were implemented with tree-bisection-reconstruction in PAUP* v4.0 [42]. To decrease computational time for each dataset, 5–10 preliminary analyses were used to estimate threshold tree lengths, and subsequent searches were allowed to run to completion saving only those trees less than or equal the observed minima of 7249 steps (A domain) and 5564 steps (KS domain) (unconstrained analysis with all taxa). Bootstrap analyses with 1000 replicates were used to estimate branch support. For Bayesian analyses, ProtTest version 2.2 [50] was used to infer a model of amino acid evolution (WAG+G and WAG+I+G for A domain and KS domain datasets, respectively) for each dataset using the Akaike Information Criterion. Bayesian analyses, implemented in MrBayes version 3.1.1 [51], consisted of two independent runs with four chains each for five million generations, sampling every 1000th generation. Convergence was estimated based on the standard deviation of split frequencies <0.01 and plots of –lnL values, which stabilized after approximately 3 million generations. The first 3 million generations were discarded as the burn-in prior to inference of majority-rule consensus trees.

### Tests of hypotheses

Constraint analyses were used to test the null hypothesis that the phylogenetic histories of the A domain and the KS domain from the hybrid NPS/PKS gene was consistent with the assumption of fungal origin. All constraint trees (Fig. 2) were prepared in MacClade [52] and loaded as constraints for MP analyses. Two analyses consisted of testing the monophyly of putatively bacterial and fungal sequences. The first analysis constrained all bacterial and all fungal sequences for each dataset as reciprocally monophyletic polytomies sister to the respective outgroup (Fig. 2A). The second analysis constrained all bacterial sequences, all previously identified HGT sequences (Table S4), and the hybrid amino acid sequences of interest as a polytomy reciprocal to a monophyletic polytomy consisting of the remaining fungal sequences (Fig. 2B). The third analysis consisted of a backbone constraint where a subset of bacterial and fungal sequences were constrained (Fig. 2C, A domain and KS domain, respectively) as reciprocal monophyletic polytomies sister to the outgroup.

Tree quality scores including tree length, consistency indices, retention indices, and topologies were compared against the unconstrained analyses of each dataset. Topological congruence of phylograms obtained from constrained vs. unconstrained analyses was assessed using Kishino-Hasegawa (KH), Templeton (TT), and Winning-Sites (WS) tests. The SH-test was implemented in RAxML [43] using the command –f h to test the likelihood scores for constrained (Fig. 2C, A domain and KS domain, respectively) and unconstrained topologies.

## Supporting Information

Figure S1 **Relationship of bacterial and fungal NRPS A domains based on the monophyly constraint of bacterial and fungal sequences as reciprocally monophyletic polytomies that together are sister to the outgroup (** **Fig. 2A** **).** Tree depicts the strict consensus from a constrained maximum parsimony analysis based on an amino acid alignment of the NRPS A domain. Taxon numbers in bold represent sequences obtained in this study.
(TIF)

Figure S2 **Relationship of bacterial and fungal NRPS A domains based on monophyly constraint of bacterial sequences, fungal sequences previously identified as having undergone HGT, and hybrid NRPS/PKS fungal sequences as a monophyletic polytomy reciprocal to a monophyletic polytomy of all other fungal sequences that together are sister to the outgroup (** **Fig. 2B** **).** Tree depicts the strict consensus from a constrained maximum parsimony analysis based on an amino acid alignment of the NRPS A domain. Taxon numbers in bold represent sequences obtained in this study.
(TIF)

Figure S3 **Relationship of bacterial and fungal NRPS A domains based on a backbone constraint of a subset of bacterial and fungal sequences as monophyletic polytomies sister to the outgroup (** **Fig. 2C, A** **domain).** Tree depicts the strict consensus from a constrained maximum parsimony analysis based on an amino acid alignment of the NRPS A domain. Taxon numbers in bold represent sequences obtained in this study.
(TIF)

Figure S4 **Relationship of bacterial and fungal PKS KS domains based on the monophyly constraint of bacterial and fungal sequences as reciprocally monophyletic polytomies that together are sister to the outgroup (** **Fig. 2A** **).** Tree depicts the strict consensus from a constrained maximum parsimony analysis based on an amino acid alignment of the PKS KS domain. Taxon numbers in bold represent sequences obtained in this study.
(TIF)

Figure S5 **Relationship of bacterial and fungal PKS KS domains based on monophyly constraint of bacterial sequences, fungal sequences previously identified as having undergone HGT, and hybrid NRPS/PKS fungal sequences as a monophyletic polytomy reciprocal to a monophyletic polytomy of all other fungal sequences that together are sister to the outgroup (** **Fig. 2B** **).** Tree depicts the strict consensus from a constrained maximum parsimony analysis based on an amino acid alignment of the PKS KS domain. Taxon numbers in bold represent sequences obtained in this study.
(TIF)

Figure S6 **Relationship of bacterial and fungal PKS KS domains based on a backbone constraint of a subset of bacterial and fungal sequences as monophyletic polytomies sister to the outgroup (** **Fig. 2C** **, KS domain).** Tree depicts the strict consensus from a constrained maximum parsimony analysis based on an amino acid alignment of the PKS KS domain. Taxon numbers in bold represent sequences obtained in this study.
(TIF)

Table S1 **Fungal species evaluated for hybrid** ***NPS7*** **/** ***PKS24*** **homolog. Isolates in bold produced PCR amplicons, confirming the presence of the hybrid** ***NPS/PKS*** **homolog in these taxa.**
(DOC)

Table S2 **NRPS A domain sequences from GenBank included in the A domain alignment.**
(DOC)

Table S3 **PKS KS domain sequences from GenBank included in the KS domain alignment.**
(DOC)

Table S4 **Previously reported horizontally transferred sequences from bacteria to fungi.**
(DOC)
