# Supplementary figures and images for: Interkingdom Gene Transfer of a Hybrid NPS/PKS from Bacteria to Filamentous Ascomycota
Source: PLoS One. 2011 Nov 29;6(11):e28231. doi: 10.1371/journal.pone.0028231 (PMC3226686; doi:10.1371/journal.pone.0028231)

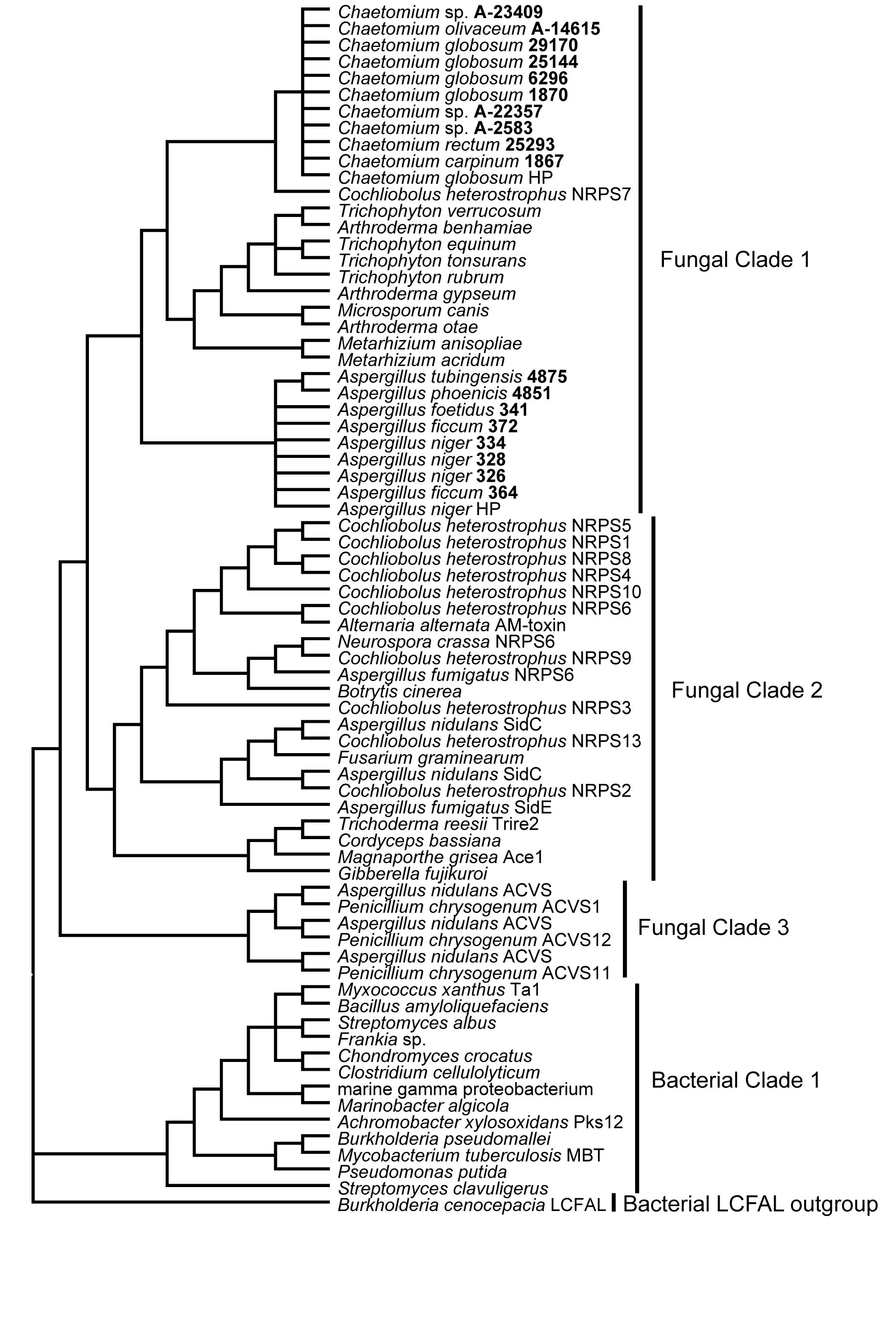

Supplement: Figure S1 — Relationship of bacterial and fungal NRPS A domains based on the monophyly constraint of bacterial and fungal sequences as reciprocally monophyletic polytomies that together are sister to the outgroup ( Fig. 2A ). Tree depicts the strict consensus from a constrained maximum parsimony analysis based on an amino acid alignment of the NRPS A domain. Taxon numbers in bold represent sequences obtained in this study. (TIF) [file pone.0028231.s001.tif]

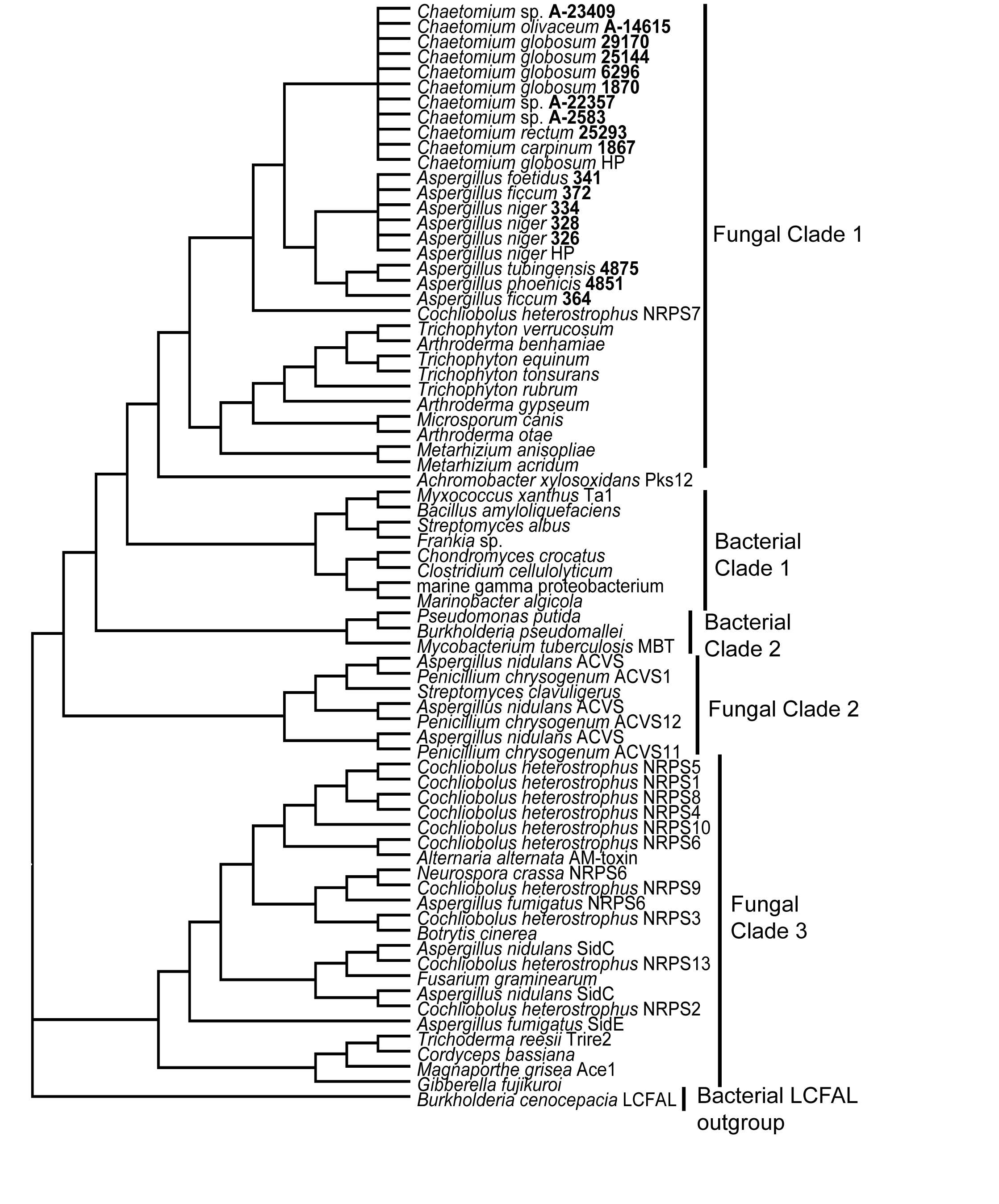

Supplement: Figure S2 — Relationship of bacterial and fungal NRPS A domains based on monophyly constraint of bacterial sequences, fungal sequences previously identified as having undergone HGT, and hybrid NRPS/PKS fungal sequences as a monophyletic polytomy reciprocal to a monophyletic polytomy of all other fungal sequences that together are sister to the outgroup ( Fig. 2B ). Tree depicts the strict consensus from a constrained maximum parsimony analysis based on an amino acid alignment of the NRPS A domain. Taxon numbers in bold represent sequences obtained in this study. (TIF) [file pone.0028231.s002.tif]

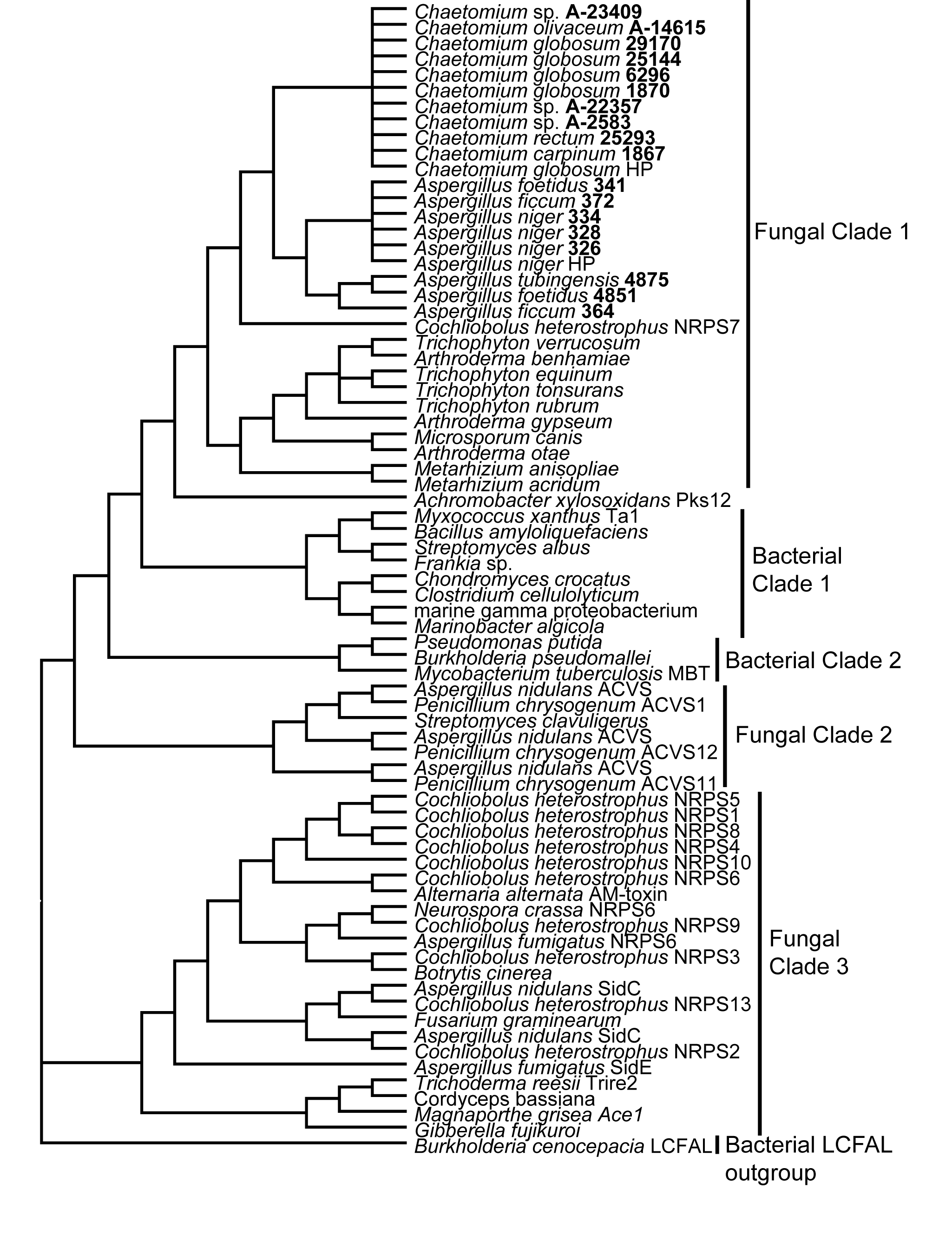

Supplement: Figure S3 — Relationship of bacterial and fungal NRPS A domains based on a backbone constraint of a subset of bacterial and fungal sequences as monophyletic polytomies sister to the outgroup ( Fig. 2C, A domain). Tree depicts the strict consensus from a constrained maximum parsimony analysis based on an amino acid alignment of the NRPS A domain. Taxon numbers in bold represent sequences obtained in this study. (TIF) [file pone.0028231.s003.tif]

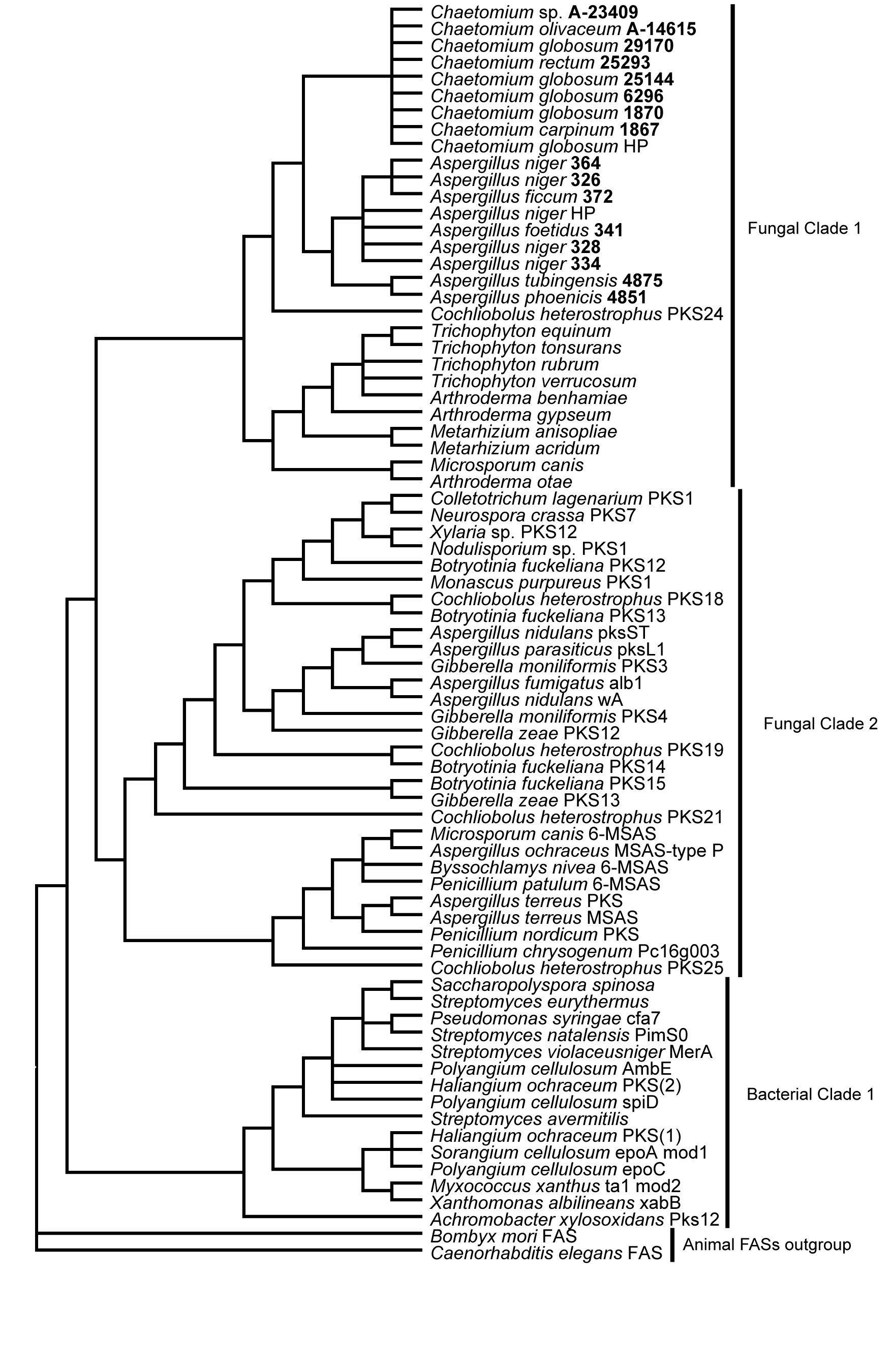

Supplement: Figure S4 — Relationship of bacterial and fungal PKS KS domains based on the monophyly constraint of bacterial and fungal sequences as reciprocally monophyletic polytomies that together are sister to the outgroup ( Fig. 2A ). Tree depicts the strict consensus from a constrained maximum parsimony analysis based on an amino acid alignment of the PKS KS domain. Taxon numbers in bold represent sequences obtained in this study. (TIF) [file pone.0028231.s004.tif]

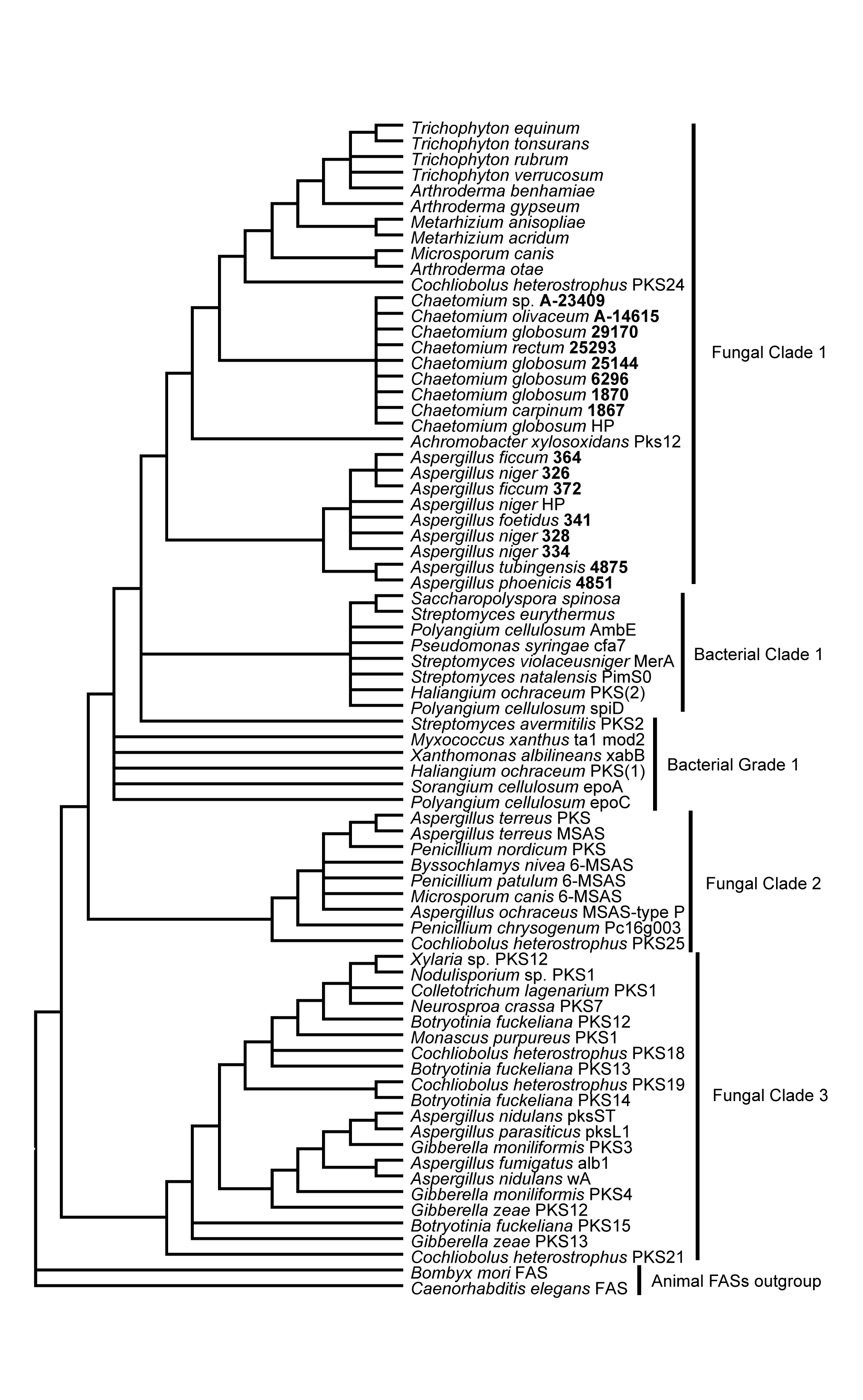

Supplement: Figure S5 — Relationship of bacterial and fungal PKS KS domains based on monophyly constraint of bacterial sequences, fungal sequences previously identified as having undergone HGT, and hybrid NRPS/PKS fungal sequences as a monophyletic polytomy reciprocal to a monophyletic polytomy of all other fungal sequences that together are sister to the outgroup ( Fig. 2B ). Tree depicts the strict consensus from a constrained maximum parsimony analysis based on an amino acid alignment of the PKS KS domain. Taxon numbers in bold represent sequences obtained in this study. (TIF) [file pone.0028231.s005.tif]

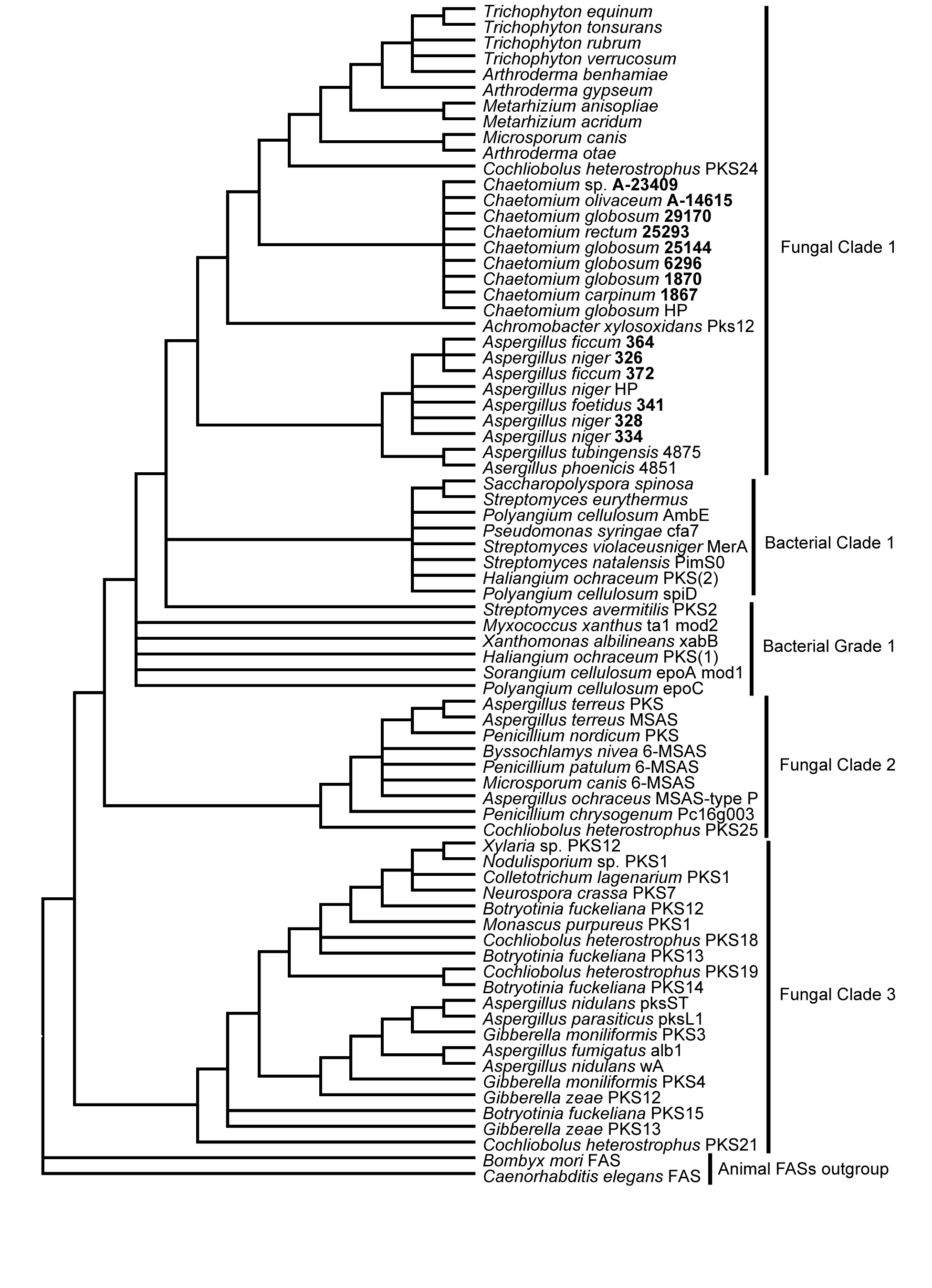

Supplement: Figure S6 — Relationship of bacterial and fungal PKS KS domains based on a backbone constraint of a subset of bacterial and fungal sequences as monophyletic polytomies sister to the outgroup ( Fig. 2C , KS domain). Tree depicts the strict consensus from a constrained maximum parsimony analysis based on an amino acid alignment of the PKS KS domain. Taxon numbers in bold represent sequences obtained in this study. (TIF) [file pone.0028231.s006.tif]
