# Supplementary material for: Interkingdom Gene Transfer of a Hybrid NPS/PKS from Bacteria to Filamentous Ascomycota
Source: PLoS One. 2011 Nov 29;6(11):e28231. doi: 10.1371/journal.pone.0028231 (PMC3226686; doi:10.1371/journal.pone.0028231)
Supplement: Table S1 — Fungal species evaluated for hybrid NPS7 / PKS24 homolog. Isolates in bold produced PCR amplicons, confirming the presence of the hybrid NPS/PKS homolog in these taxa. (DOC) [file pone.0028231.s007.doc]

**Table S1.** Fungal species evaluated for the hybrid *NPS*/*PKS* homolog. Isolates in bold produced PCR amplicons, confirming the presence of the hybrid *NPS*/*PKS* homolog in these taxa.

| ***Aspergillus* Section *Nigri* species** | | ***Chaetomium* species** | | | |
| --- | --- | --- | --- | --- | --- |
| NRRL 358 | *A. aculeatus* | NRRL 25279 | *Ch. amygdalisporum* | NRRL 2174 | *Ch. indicum* |
| NRRL 5094 | *A. aculeatus* | NRRL 26044 | *Ch. anguipilium* | NRRL 25294 | *Ch. indicum* |
| NRRL 356 | *A. awamori* | NRRL 25245 | *Ch. angustispirale* | NRRL 25241 | *Ch. irregulare* |
| NRRL 4948 | *A. awamori* | NRRL A-27396 | *Ch. apiculatum* | NRRL A-7818 | *Ch. microcephalum* |
| NRRL 35542 | *A. brasiliensis* | NRRL 25280 | *Ch. atrobrunneum* | NRRL A-14853 | *Ch. mollicellum* |
| NRRL 67 | *A. carbonarius* | NRRL 26045 | *Ch. aureum* | NRRL 25285 | *Ch. nigricolor* |
| NRRL 369 | *A. carbonarius* | NRRL 26046 | *Ch. barilochense* | NRRL 25661 | *Ch. ochraceum* |
| NRRL 4849 | *A. carbonarius* | NRRL 26047 | *Ch. bostrychodes* | **NRRL A-14615** | ***Ch. olivaceum*** |
| NRRL 5120 | *A. ellipticus* | NRRL 26048 | *Ch. bostrychodes* | **NRRL 25293** | ***Ch. rectum*** |
| **NRRL 364** | ***A. ficcum*** | NRRL 26049 | *Ch. brasiliense* | NRRL 2195 | *Ch. reflexum* |
| **NRRL 372** | ***A. ficcum*** | NRRL 26050 | *Ch. brasiliense* | NRRL 25635 | *Ch. reticulopilum* |
| NRRL 337 | *A. foetidus* | NRRL 26051 | *Ch. brasiliense* | NRRL A-25809 | *Ch. spinosum* |
| **NRRL 341** | ***A. foetidus*** | **NRRL 1867** | ***Ch. carpinum*** | NRRL A-10898 | *Ch. strumarium* |
| NRRL 4747 | *A. heteromorphus* | NRRL 26052 | *Ch. carinthiacum* | NRRL 25284 | *Ch. subglobosum* |
| NRRL 35645 | *A. ibericus* | NRRL A-27406 | *Ch. causiaeformis* | NRRL A-17074 | *Ch. subspirale* |
| NRRL 360 | *A. japonicus* | NRRL 26053 | *Ch. circinatum* | NRRL 25289 | *Ch. tennissimum* |
| NRRL 2053 | *A. japonicus* | NRRL 25292 | *Ch. coarctatum* | NRRL A-25811 | *Ch. tortile* |
| **NRRL 326** | ***A. niger*** | NRRL 2320 | *Ch. cochliodes* | NRRL 6117 | *Ch. trilaterale* |
| **NRRL 328** | ***A. niger*** | NRRL 26055 | *Ch. convulutum* | NRRL 25240 | *Ch. trilaterale* var. *chiversii* |
| NRRL 330 | *A. niger* | NRRL 26056 | *Ch. crispatum* | NRRL 6547 | *Ch. udagawae* |
| **NRRL 334** | ***A. niger*** | NRRL 26058 | *Ch. cupreum* | NRRL A-27347 | *Ch. venezuelense* |
| NRRL 363 | *A. niger* | NRRL 26059 | *Ch. cupreum* | NRRL 25287 | *Ch. virescens* |
| NRRL 3536 | *A. niger* | NRRL 1606 | *Ch. dolichotrichum* | **NRRL A-2583** | ***Chaetomium* sp.** |
| NRRL 365 | *A. phoenicis* | NRRL 2171 | *Ch. elatum* | NRRL A-2712 | *Chaetomium* sp. |
| NRRL 593 | *A. phoenicis* | NRRL 2192 | *Ch. erectum* | NRRL A-3114 | *Chaetomium* sp. |
| NRRL 4750 | *A. phoenicis* | NRRL 2172 | *Ch. funicolum* | NRRL A-3666 | *Chaetomium* sp. |
| **NRRL 4851** | ***A. phoenicis*** | NRRL A-28307 | *Ch. funicolum* | NRRL A-7797 | *Chaetomium* sp. |
| **NRRL 4875** | ***A. tubingensis*** | NRRL 25244 | *Ch. fusum* | NRRL A-21205 | *Chaetomium* sp. |
| ***Aspergillus* Section *Flavi* species** | | NRRL 25283 | *Ch. gangligerum* | **NRRL A-22357** | ***Chaetomium* sp.** |
| NRRL 517 | *A. avenaceus* | NRRL 25291 | *Ch. globosporum* | **NRRL A-23409** | ***Chaetomium* sp.** |
| NRRL 26010 | *A. bombycis* | **NRRL 1870** | ***Ch. globosum*** | NRRL A-28056 | *Chaetomium* sp. |
| NRRL 25528 | *A. caelatus* | NRRL 2173 | *Ch. globosum* | NRRL A-28306 | *Chaetomium* sp. |
| NRRL 1957 | *A. flavus* | **NRRL 6296** | ***Ch. globosum*** |  |  |
| NRRL 3648 | *A. lanosus* | **NRRL 25144** | ***Ch. globosum*** |  |  |
| NRRL 13137 | *A. nomius* | **NRRL 29170** | ***Ch. globosum*** |  |  |
| NRRL 502 | *A. parasiticus* | NRRL A-28064 | *Ch. globosum* |  |  |
| NRRL 25517 | *A. pseudotamarii* | NRRL 25243 | *Ch. gracile* |  |  |
| NRRL 4998 | *A. subolivaceus* | NRRL 26054 | *Ch. hissarense* |  |  |
| NRRL 20818 | *A. tamarii* | NRRL 25242 | *Ch. homopilatum* |  |  |
