# Supplementary material for: Interkingdom Gene Transfer of a Hybrid NPS/PKS from Bacteria to Filamentous Ascomycota
Source: PLoS One. 2011 Nov 29;6(11):e28231. doi: 10.1371/journal.pone.0028231 (PMC3226686; doi:10.1371/journal.pone.0028231)
Supplement: Table S2 — NRPS A domain sequences from GenBank included in the A domain alignment. (DOC) [file pone.0028231.s008.doc]

**Table S2.** Taxa, phylum or class, protein, and accession number for sequences from GenBank used in phylogenetic analyses of NRPS A domain.

| **a. Bacteria** | **Phylum** | **Protein (module)** | **Accession #** |
| --- | --- | --- | --- |
| *Frankia* sp. | Actinobacteria | AA adenylation | YP_481079 |
| *Mycobacterium tuberculosis* | Actinobacteria | peptide synthetase | ZP_03425674 |
| *Streptomyces albus* | Actinobacteria | ozmO | ABS90476 |
| *Streptomyces clavuligerus* | Actinobacteria | ACVS | ZP_05002819 |
| *Bacillus amyloliquefaciens* | Firmicutes | BaeJ | YP_001421292 |
| *Bacillus cereus* | Firmicutes | glycine-AMP ligase | BC2306 |
| *Clostridium cellulolyticum* | Firmicutes | AA adenylation | YP_002506647 |
| *Achromobacter xylosoxidans* | Proteobacteria | Pks12 | Top of Form  EGP48546Bottom of Form |
| *Burkholderia cenocepacia* | Proteobacteria | LCFAL | YP_001763426 |
| *Burkholderia pseudomallei* | Proteobacteria | NRPS | ZP_03795572 |
| *Chondromyces crocatus* | Proteobacteria | PKS/NRPS | CAQ18839 |
| marine gamma proteobacterium | Proteobacteria | peptide synthetase | ZP_01618156 |
| *Marinobacter algicola* | Proteobacteria | peptide synthetase | ZP_01892961 |
| *Myxococcus xanthus* | Proteobacteria | Ta1 | CAB38084 |
| *Pseudomonas putida* | Proteobacteria | non-ribosomal siderophore peptide synthetase | NP_746336 |
|  |  |  |  |
| **b. Fungi** | **Class** | **Protein (module)** | **Accession #** |
| *Alternaria alternata* | Dothideomycetes | AM-toxin | AAF01762 |
| *Cochliobolus heterostrophus* | Dothideomycetes | NRPS1 | AAX09983 |
| *Cochliobolus heterostrophus* | Dothideomycetes | NRPS2 | AAX09984 |
| *Cochliobolus heterostrophus* | Dothideomycetes | NRPS3 | AAX09985 |
| *Cochliobolus heterostrophus* | Dothideomycetes | NRPS4 | AAX09986 |
| *Cochliobolus heterostrophus* | Dothideomycetes | NRPS5 | AAX09987 |
| *Cochliobolus heterostrophus* | Dothideomycetes | NRPS6 | AAX09988 |
| *Cochliobolus heterostrophus* | Dothideomycetes | NRPS7/PKS24 | AAR90278 |
| *Cochliobolus heterostrophus* | Dothideomycetes | NRPS8 | AAX09990 |
| *Cochliobolus heterostrophus* | Dothideomycetes | NRPS9 | AAX09991 |
| *Cochliobolus heterostrophus* | Dothideomycetes | NRPS10 | AAX09992 |
| *Cochliobolus heterostrophus* | Dothideomycetes | NPRS13 | AY884198 |
| *Arthroderma benhamiae* | Eurotiomycetes | hypothetical protein | XP_003014124 |
| *Arthroderma gypseum* | Eurotiomycetes | L-aminoadipate-semialdehyde dehydrogenase | XP_003176907 |
| *Arthroderma otae* | Eurotiomycetes | NRPS | XP_002850891 |
| *Aspergillus fumigatus* | Eurotiomycetes | NRPS6 | AAX11421 |
| *Aspergillus fumigatus* | Eurotiomycetes | SidE | XP_748654 |
| *Aspergillus nidulans* | Eurotiomycetes | ACVS | AN2621.4 |
| *Aspergillus nidulans* | Eurotiomycetes | ACVS | AN2621.4 |
| *Aspergillus nidulans* | Eurotiomycetes | ACVS | AN2621.4 |
| *Aspergillus nidulans* | Eurotiomycetes | SidC | AAP56239 |
| *Aspergillus niger* | Eurotiomycetes | hypothetical protein | CAK42046 |
| *Microsporum canis* | Eurotiomycetes | NRPS | EEQ28107 |
| *Penicillium chrysogenum* | Eurotiomycetes | ACVS1 | ABR12615 |
| *Penicillium chrysogenum* | Eurotiomycetes | ACVS11 | ABA70582 |
| *Penicillium chrysogenum* | Eurotiomycetes | ACVS12 | ABR12615 |
| *Trichophyton equinum* | Eurotiomycetes | NRPS | EGE01982 |
| *Trichophyton rubrum* | Eurotiomycetes | NRPS | XP_003238870 |
| *Trichophyton tosurans* | Eurotiomycetes | NRPS | EGD97139 |
| *Trichophyton verrucosum* | Eurotiomycetes | hypothetical protein | XP_003020763 |
| *Botrytis cinera* | Leotiomycetes | hypothetical protein | XP_001556764 |
| *Cordyceps bassiana* | Sordariomycetes | NRPS; tenellin PKS | CAL69597 |
| *Fusarium graminearum* | Sordariomycetes | NRPS | XP_391202 |
| *Chaetomium globosum* | Sordariomycetes | hypothetical protein | XP_001224327 |
| *Gibberella fujikuroi* | Sordariomycetes | PKS | AAT28740 |
| *Gibberella zeae* | Sordariomycetes | acyl CoA ligase-like protein | AAP12366 |
| *Magnaporthe grisea* | Sordariomycetes | Ace1 | CAG28798 |
| *Metarhizium acridum* | Sordariomycetes | NRPS | EFY84397 |
| *Metarhizium anisopliae* | Sordariomycetes | NRPS | EFY95969 |
| *Neurospora crassa* | Sordariomycetes | hypothetical protein | XP_963411 |
| *Trichoderma reesei* | Sordariomycetes | PKS/NRPS | Trire2_58285 |
