# Supplementary material for: Interkingdom Gene Transfer of a Hybrid NPS/PKS from Bacteria to Filamentous Ascomycota
Source: PLoS One. 2011 Nov 29;6(11):e28231. doi: 10.1371/journal.pone.0028231 (PMC3226686; doi:10.1371/journal.pone.0028231)
Supplement: Table S3 — PKS KS domain sequences from GenBank included in the KS domain alignment. (DOC) [file pone.0028231.s009.doc]

**Table S3.** Taxa, phylum or class, protein, and accession number for sequences from GenBank used in phylogenetic analyses of PKS KS domain.

| **a. Bacteria** | **Phylum** | **Protein (module)** | **Accession #** |
| --- | --- | --- | --- |
| *Saccharopolyspora spinosa* | Actinobacteria | ObsA | AAS00419 |
| *Streptomyces avermitilis* | Actinobacteria | PKS2 | NP_822727 |
| *Streptomyces eurythermus* | Actinobacteria | angI | ABV49610 |
| *Streptomyces natalensis* | Actinobacteria | PimS0 | CAB41040 |
| *Streptomyces violaceusniger* | Actinobacteria | MerA | ABJ97437 |
| *Achromobacter xylosoxidans* | Proteobacteria | Pks12 | Top of Form  EGP48546Bottom of Form |
| *Haliangium ochraceum* | Proteobacteria | PKS family protein (1) | ZP_03880774 |
| *Haliangium ochraceum* | Proteobacteria | PKS family protein (2) | ZP_03880776 |
| *Myxococcus xanthus* | Proteobacteria | ta1 mod2 | CAB38084 |
| *Polyangium cellulosum* | Proteobacteria | AmbE | ABK32259 |
| *Polyangium cellulosum* | Proteobacteria | epoC | AAF26921 |
| *Polyangium cellulosum* | Proteobacteria | spiD | CAL58681 |
| *Pseudomonas syringae* | Proteobacteria | cfa7 | AAD03048 |
| *Sorangium cellulosum* | Proteobacteria | epoA mod1 | AAF62880 |
| *Xanthomonas albilineans* | Proteobacteria | xabB | CAE52339 |
|  |  |  |  |
| **b. Fungi** | **Class** | **Protein (module)** | **Accession #** |
| *Cochliobolus heterostrophus* | Dothideomycetes | NRPS7/PKS24 | AAR90278 |
| *Cochliobolus heterostrophus* | Dothideomycetes | PKS15 | AAR90269 |
| *Cochliobolus heterostrophus* | Dothideomycetes | PKS18 | AAR90272 |
| *Cochliobolus heterostrophus* | Dothideomycetes | PKS19 | AAR90273 |
| *Cochliobolus heterostrophus* | Dothideomycetes | PKS21 | AAR90275 |
| *Cochliobolus heterostrophus* | Dothideomycetes | PKS25 | AAR90279 |
| *Byssochlamys nivea* | Eurotiomycetes | 6-MSAS | AAK48943 |
| *Arthroderma benhamiae* | Eurotiomycetes | hypothetical protein | XP_003014124 |
| *Arthroderma gypseum* | Eurotiomycetes | L-aminoadipate-semialdehyde dehydrogenase | XP_003176907 |
| *Arthroderma otae* | Eurotiomycetes | PKS | XP_002850891 |
| *Aspergillus fumigatus* | Eurotiomycetes | alb1 | AAC39471 |
| *Aspergillus nidulans* | Eurotiomycetes | wA | CAA46695 |
| *Aspergillus nidulans* | Eurotiomycetes | pksST | AAA81586 |
| *Aspergillus niger* | Eurotiomycetes | hypothetical protein | CAK42046 |
| *Aspergillus ochraceus* | Eurotiomycetes | MSAS-type PKS | AAS98200 |
| *Aspergillus parasiticus* | Eurotiomycetes | pksL1 | AAC41675 |
| *Aspergillus terreus* | Eurotiomycetes | 6-MSAS | BAA20102 |
| *Aspergillus terreus* | Eurotiomycetes | pksM | AAC49814 |
| *Microsporum canis* | Eurotiomycetes | PKS | EEQ28107 |
| *Microsporum canis* | Eurotiomycetes | 6-MSAS | EEQ29781 |
| *Monascus purpureus* | Eurotiomycetes | PKS1 | CAC94008 |
| *Penicillium chrysogenum* | Eurotiomycetes | Pc16g00370 | XP_002560460 |
| *Penicillium nordicum* | Eurotiomycetes | ochratoxin A PKS | AAP33839 |
| *Penicillium patulum* | Eurotiomycetes | 6-MSAS | CAA39295 |
| *Trichophyton equinum* | Eurotiomycetes | PKS | EGE01982 |
| *Trichophyton rubrum* | Eurotiomycetes | PKS | XP_003238870 |
| *Trichophyton tosurans* | Eurotiomycetes | PKS | EGD97139 |
| *Trichophyton verrucosum* | Eurotiomycetes | hypothetical protein | XP_003020763 |
| *Botryotinia fuckeliana* | Leotiomycetes | PKS12 | AAR90248 |
| *Botryotinia fuckeliana* | Leotiomycetes | PKS13 | AAR90249 |
| *Botryotinia fuckeliana* | Leotiomycetes | PKS14 | AAR90250 |
| *Botryotinia fuckeliana* | Leotiomycetes | PKS15 | AAR90251 |
| *Chaetomium globosum* | Sordariomycetes | hypothetical protein | XP_001224327 |
| *Colletotrichum lagenarium* | Sordariomycetes | PKS1 | BAA18956 |
| *Gibberella moniliformis* | Sordariomycetes | PKS3 | AAR92210 |
| *Gibberella moniliformis* | Sordariomycetes | PKS4 | AAR92211 |
| *Gibberella zeae* | Sordariomycetes | PKS12 | AAS57296 |
| *Gibberella zeae* | Sordariomycetes | PKS13 | ABB90282 |
| *Metarhizium acridum* | Sordariomycetes | PKS | EFY84397 |
| *Metarhizium anisopliae* | Sordariomycetes | PKS | EFY95969 |
| *Neurospora crassa* | Sordariomycetes | PKS7 | XP_322886 |
| *Nodulisporium* sp. | Sordariomycetes | PKS1 | AAD38786 |
| *Xylaria* sp. | Sordariomycetes | PKS12 | AAM9354 |
|  |  |  |  |
| **c. Animal** | **Phylum** | **Protein (module)** | **Accession #** |
| *Bombyx mori* | Arthropoda | FAS | NP_001037478 |
| *Caenorhabditis elegans* | Nematoda | FAS | NP_492417 |
