# Supplementary material for: Interkingdom Gene Transfer of a Hybrid NPS/PKS from Bacteria to Filamentous Ascomycota
Source: PLoS One. 2011 Nov 29;6(11):e28231. doi: 10.1371/journal.pone.0028231 (PMC3226686; doi:10.1371/journal.pone.0028231)
Supplement: Table S4 — Previously reported horizontally transferred sequences from bacteria to fungi. (DOC) [file pone.0028231.s010.doc]

**Table S4.** Previously reported horizontally transferred sequences from bacteria to fungi.

| **a. Fungal NRPS A domains** | **Class** | **Protein (module)** | **Accession #** |
| --- | --- | --- | --- |
| *Aspergillus nidulans* | Eurotiomycetes | ACVS | AN2621.4^a^ |
| *Aspergillus nidulans* | Eurotiomycetes | ACVS | AN2621.4 ^a^ |
| *Aspergillus nidulans* | Eurotiomycetes | ACVS | AN2621.4 ^a^ |
| *Penicillium chrysogenum* | Eurotiomycetes | ACVS1 | ABR12615 ^a^ |
| *Penicillium chrysogenum* | Eurotiomycetes | ACVS11 | ABA70582 ^a^ |
| *Penicillium chrysogenum* | Eurotiomycetes | ACVS12 | ABR12615 ^a^ |
|  |  |  |  |
| **b. Fungal PKS KS domains** | **Class** | **Protein (module)** | **Accession #** |
| *Aspergillus ochraceus* | Eurotiomycetes | MSAS-type PKS | AAS98200 ^b^ |
| *Aspergillus terreus* | Eurotiomycetes | pksM | AAC49814 ^b^ |
| *Aspergillus terreus* | Eurotiomycetes | 6-MSAS | BAA20102 ^b^ |
| *Byssochlamys nivea* | Eurotiomycetes | 6-MSAS | AAK48943 ^b^ |
| *Microsporum canis* | Eurotiomycetes | 6-MSAS | EEQ29781 ^b^ |
| *Penicillium chrysogenum* | Eurotiomycetes | Pc16g00370 | XP_002560460 ^c^ |
| *Penicillium nordicum* | Eurotiomycetes | ochratoxin A PKS | AAP33839 ^c^ |
| *Penicillium patulum* | Eurotiomycetes | 6-MSAS | CAA39295 ^b^ |
| *Cochliobolus heterostrophus* | Dothideomycetes | PKS25 | AAR90279 ^b^ |

^a^  Identified previously as being transferred from bacteria to fungi via HGT [41].

^b^ Identified previously as being transferred from bacteria to fungi via HGT [24].

^c^ Identified in this study as having been putatively transferred from bacteria to fungi via HGT.
